# Influence of emotional intelligence on the clinical ability of nursing interns: a structural equation model

**DOI:** 10.1186/s12912-022-00933-y

**Published:** 2022-06-13

**Authors:** Shuangting Dou, Chenyan Han, Conghong Li, Xiaoxin Liu, Wanling Gan

**Affiliations:** 1 Department of Nursing, Shanghai Chest Hospital, Shanghai Jiao Tong University, 241 Huai Hai West Road, Xuhui District, Shanghai, 200030 China; 2 Teaching and Research Department of Clinical Nursing, Shanghai Jiao Tong University School of Nursing, 227 South Chong Qing Road, Building 1, Room 209, Huangpu District, Shanghai, 200025 China

**Keywords:** EI, Clinical ability, Nursing interns, Nursing education

## Abstract

**Background:**

Internship is a critical period during which nursing students develop clinical skills and establish professional attitudes. Requirements for nursing interns are evolving with the development of medicine and the transformation of teaching models. The emotional intelligence (EI) of nursing students has an influence on their clinical performance. This study aimed to investigate the impact of EI on the clinical ability of nursing interns.

**Methods:**

A cross-sectional observational study was designed to include nursing students interning in a tertiary hospital in Shanghai, China from April 1, 2019 to April 30, 2020 (*N* = 310). Chinese versions of the EI scale (EIS) and holistic clinical assessment tool (HCAT) were used to measure the EI and clinical ability of interns, respectively. Pearson’s correlation coefficient was utilized to determine the correlation between EI and clinical ability. Multiple linear regression analysis was performed to further explore the influence of EI on clinical ability, and the structural equation model (SEM) was used for multivariate path analysis.

**Results:**

The mean EI and clinical ability scores of interns were 125.17 ± 14.98 and 97.91 ± 19.55, respectively, indicating an upper-moderate level in both aspects. EI scores were correlated positively with clinical ability ones (*R *= 0.534, *p* < 0.05). Multivariate path analysis showed that “managing emotions” and “facilitating thought” of EI branches have direct effects on clinical ability. Furthermore, the type of school, family financial state and the knowledge of EI indirectly influence clinical ability through their impact on “managing emotions” and “facilitating thought”.

**Conclusions:**

EI is essential to enhancing the clinical ability of nursing students. EI training should focus on facilitating thought and managing emotions. It is also necessary for educators to consider the context of nursing students and the characteristics of schools.

**Supplementary Information:**

The online version contains supplementary material available at 10.1186/s12912-022-00933-y.

## Background

Emotion is a key motivator for nursing decision-making and action, and every nursing intervention is affected by the emotional ability of nurses which is known as emotional intelligence (EI). Referring to psychosocial ability capable of changing both academic and workplace outcomes [1], EI can be cultivated and improved through education and training, and might exert an impact on the learning quality, ethical decision-making and critical thinking of nursing students, evidence and knowledge use in practice, the quality of patient care and patient outcomes [2, 3]. Nursing internship is a key period for nursing students to gain clinical skills and develop clinical ability. Therefore, it is of importance to evaluate the EI of nursing interns and further explore its relationship with clinical ability.

In the educational field, EI originally proposed by Salovey and Mayer in 1990 is defined as social intelligence that controls one's feelings and emotions and uses information as a sub-element to guide one's own thoughts and actions [4]. After several revisions, it now means social ability involving the ability to accurately perceive, access and generate emotions so as to help thought and understand emotions and emotional knowledge, and reflectively regulate emotions in order to promote emotional and intellectual growth [4]. EI has two models: trait- or ability-based EI. Trait EI is a collection of emotional self-perceptions located at the lower levels of personality hierarchies and integrates the affective aspects of personality [5]. As a representative model of ability EI, the four-branch model of Mayer operationally defines EI to comprise the following: a) correctly perceiving one's own and other's feelings (perceiving emotions), b) using emotional information to advance thinking (facilitating thought), c) analyzing emotions and their relationships, predicting or understanding possible consequences (understanding emotions), and (d) avoiding emotions and reevaluating value for the sake of self-calmness (managing emotions) [4]. According to this model, the use of emotions in thinking and decision making can be a form of intelligence. A good combination of emotions and cognition can facilitate decision-making, manage emotions, improve relationships, and ultimately lead to smarter decisions [6]. Clinical decision-making is a primary part of nursing practice [7], and decision-making is an ultimate skill for nurses. In view of this, the ability EI model was adopted as the theoretical basis.

A lot of EI measurement tools are based on the ability model, among which the Mayer-Salovey-Caruso EI Test (MSCEIT) is a task-oriented tool similar to the general intelligence quotient (IQ) test. Consisting of 141 items, MSCEIT takes 30-45 minutes to complete [8]. In addition, a self-report measure of EI (SEI) based on the ability model was developed by Schutte (1998), which is composed of 33 items and has been rigorously tested for reliability and validity. Moreover, it is characterized by the elimination of duplication with cognitive abilities and personalities like language and mathematics ability.

Existing studies have shown that EI is important for the nursing profession, including developing therapeutic nurse-patient relationships, providing high-quality nursing and effectively using the consulting role of nurses [9]. Additionally, EI also helps nurses to understand and calmly cope with the complexity of interpersonal relationships [10], adjust themselves, relieve stress, and improve work efficiency in the face of environmental changes and stress overload [11].

Clinical ability is a skill that every nurse must possess—a combination of clinical expertise, attitude and ability [12]. The term clinical ability is also commonly used. A clinical internship is an important way for nursing students to test and improve clinical ability which is considered the ability of students to integrate knowledge, skills, attitudes and values into specific nursing practices [4, 12]. The most important goal of clinical education in nursing is to improve the practical skills and clinical competencies of nursing students. High clinical ability improves not only the medical experience of patients but also the professional identity and sense of accomplishment of nursing students [9].

To date, a large body of research has been committed to exploring factors that may influence the clinical ability of nursing students, including EI. It has been proven that EI can predict the success of nursing students in clinical practice and academic ability [13, 14]. In previous studies, it has been indicated that EI is significantly and positively correlated with the overall clinical ability [15], problem-solving skills, critical thinking, empathy and clinical communication, positive work attitudes and self-efficacy [3, 16] to improve the ability of nurses to meet clinical needs. A study conducted in mainland of China also showed that the EI of nursing students can predict their clinical care ability [17].

Although multiple studies have demonstrated the relationship between the EI and the clinical ability of nursing interns, the path of EI affecting the clinical ability of interns, the influence weight of four branches of ability, and the role of the demographic background of interns in the process are unclear. Particularly in mainland of China, research in this area is limited.

Therefore, this study was aimed at investigating the influence of EI on the clinical ability of nursing interns, analyzing the correlation between the two, exploring the impact of background information, and providing a reference for basic nursing education. The following specific research questions were addressed:

- 1. What are the score levels of EI and self-reported clinical ability in the nursing intern sample?
- 2. Are there significant correlations between EI and self-reported clinical ability scores?
- 3. Are there significant differences demonstrated in self-reported clinical ability scores between genders or other background or educational variables?
- 4. How do EI and other background variables predict self-reported clinical ability?

## Methods

### Design, setting and participants

In this study, a cross-sectional investigation was conducted to analyze the influence of EI on the clinical ability of nursing students. Nursing students who practiced in a Grade A hospital in Shanghai, China from April 1, 2019 to April 30, 2020 were screened. Inclusion criteria were: a) Full-time nursing students who have not graduated, b) Nursing students receiving college or undergraduate education, c) Nursing students with continuous clinical practice lasting longer than one month, and d) Nursing students able to provide informed consent. Students who had participated in the supplementary internship would be excluded. Before the study, the consent of the Nursing Department of the surveyed hospital was obtained, and then the significance, purpose and content of the study were introduced to nursing students. After giving informed consent, nursing students participated in the questionnaire survey and filled out questionnaires anonymously. A total of 340 nursing students were sampled, among which 320 were recovered. Questionnaire quality control and rejection criteria: Questionnaires with incomplete information, all choosing the same answer and 10% of items for missing values were regarded as invalid questionnaires to be rejected. After the exclusion of 10 invalid questionnaires, the final sample size included in the study was 310.

### Instruments

#### Demographic data

A general information questionnaire includes six items: gender, the type of educational system and school, internship duration, previous knowledge of EI as well as internship months and family economy. In this study, the family economy was divided into two categories which were selected by research objects according to their own family income. The previous knowledge of EI was reported with “yes” or “no”. The subjects of this study are all seniors aged from 21 to 23.

EI was measured with the Chinese version of the EI scale (EIS) compiled by American psychologist Schutte et al. [18] according to the EI theory of Salovey and Mayer and translated by Chinese psychologist and Professor Wang Caikang [19]. The score ranges from 33 to 165 points, with a total of 33 items. The four-branch ability model was distinguished among four problem-solving areas necessary to carry out emotional reasoning: The first was (a) emotion perception which was considered to be computationally most basic. Then, this work proceeded through the increasingly integrated and more cognitively complex areas of (b) promoting emotional thinking, (c) understanding and (d) management (Mayer & Salovey, 1997). (These problem-solving areas were referred to as branches after the line drawing in the original diagram.) Likert 5-level scoring method was adopted in the scale (scores 1, 2, 3, 4 and 5 = disagreement, less disagreement, uncertainty, more agreement and agreement, respectively). Items 5, 28 and 33 were scored in reverse. The higher the score was, the higher EI would be. The research of Liu Shaopeng et al. [20] showed that the reliability of each dimension and total scale in the Chinese version of EIS is 0.735, 0.815, 0.801, 0.790 and 0.821, respectively, indicating good reliability and validity. The attribution and factor composition structure of each item in the scale were consistent with the design concept of the original scale, suggesting that the Chinese version of EIS demonstrates good structural validity and has also achieved good reliability and validity in nursing professional tests. In this study, the Cronbach α of the Chinese version of EIS is 0.892, which is completely suitable for studying the EI of nursing students.

Clinical ability was measured with the Chinese version of the holistic clinical assessment tool (HCAT) developed by Singaporean scholars Wu et al. [21] and translated by Professor Yu Miao [22] of China. The Cronbach's α coefficient, test-retest reliability and average content validity of the scale are 0.965, 0.928 and 0.972, respectively. [21] It was proven that HCAT is a reliable and effective clinical evaluation tool for evaluating the clinical ability of nursing students as a whole. The clinical ability score of the scale ranges from 36 to 144 points, with a total of 36 items, including four dimensions: specialty, legal and ethical practice, clinical care, leadership and nursing management, and professional development. The scale adopted the Likert 4-level scoring method (1, 2, 3, and 4 points = unqualified, qualified, skilled and excellent, respectively). The higher the score was, the higher the clinical ability level would be. In this study, Cronbach's coefficient of the Chinese version of HCAT is 0.974.

### Procedure and statistical analysis

Statistical analysis was performed using Statistical Product Service Solutions (SPSS) software (version 24.0) (International Business Machines Corporation (IBM) SPAA, Chicago, Illinois (IL), the United States of America (USA)). Frequency and percentage (%) were used to describe categorical data. Mean and standard deviation was employed to describe continuous data. Two independent sample t-tests and a one-way analysis of variance (ANOVA) were applied to compare the measurement data between groups. The comparison of counting data groups was analyzed by the X^2^ test. Pearson correlation analysis was performed to explore the correlation between the EI and clinical ability of nursing students, with *P* < 0.05 indicating that the difference had statistical significance. To further explore the influence of EI on clinical ability, this paper divided research variables into three categories, namely dependent, independent and intermediary variables. Independent variables were items in general data, dependent variables referred to clinical ability, and each dimension of EI was used as the mediating variable. AMOS from SPSS (version 24.0) was used to build a multivariate path analysis model which was verified. According to the test results, the model was debugged many times to reach the adaptation model.

## Results

### Participants and descriptive statistics

All the participants are 21 to 23 years old. The results of the general data are shown in Table 1. Of these participants in the survey, 33.2% studied in public schools and 66.8% in private ones; 16.5% and 83.5% had relatively tight and affluent family economies, respectively; 72.3% possessed some knowledge of EI. The type of school, family economy and the knowledge of EI had an influence on the emotional intelligence level of the participants (*P* < 0.05), and the educational system of nursing students affected their clinical ability (*P* < 0.05).

**Table 1:** Analysis of general data of nursing students and their influence (*N *= 310)

**Variable**		**n (%)**	**Emotional intelligence**			**Clinical ability**		
			**Mean (SD)**	**T/X**^**2**^**/F**	**P**	**Mean (SD)**	**T/X**^**2**^**/F**	**P**
educational system	Junior college	150(48.4)	124.73(14.18)	5.10	0.61	95.16(16.91)	5.80	0.02
	Undergraduate	160 (51.6)	125.58(15.71)			100.48(21.48)		
School type	public school	103 (33.2)	127.14(14.32)	1.34	0.04	100.32(20.95)	1.45	0.23
	private school	207 (66.8)	120.11(18.39)			97.50(20.68)		
Family economy	above average	51 (16.5)	120.54(18.28)	5.92	0.02	98.57(21.18)	0.07	0.79
	below averages	259 (83.5)	126.08(14.09)			97.77(19.26)		
EI previous knowledge	Yes	224 (72.3)	121.00(19.51)	9.50	0.00	97.83(17.57)	0.01	0.92
	No	86 (27.7)	126.78(12.50)			98.08(24.08)		

^*^P < 0.05, the difference was statistically significant

### Correlation analysis

The average total scores of EI and clinical ability of nursing students are 125.17 ± 14.98 and 97.91 ± 19.55, respectively. Pearson correlation analysis showed that the EI and clinical ability of nursing students exhibited a medium correlation. Besides, each dimension of EI showed a moderate correlation with clinical ability. Meanwhile, a low correlation existed between the internship duration and the clinical ability of nursing students, as shown in Table 2 for details.

**Table 2:** The correlation of clinical ability and other variables (*N *= 310)

**Dimension**	**Mean (SD)**	**r**
total EI Score	125.17(14.98)	0.534**
emotion perception	30.19(3.94)	0.432**
promote emotional thinking	30.31(4.37)	0.446**
emotional understanding	29.58(3.74)	0.474**
emotional management	35.09(4.91)	0.524**
Internship months		0.161**
Total score of clinical ability	97.91(19.55)	

^**^P < 0.01, the difference was statistically significant

### Pathway analysis of the clinical ability

After debugging, the results are shown in Table 3. The significance probability value of the model is *P *= 0. 173 > 0.05, indicating that the model can adapt to the sample data. Chi-square degree of freedom ratio 1.383, the goodness of fit index (GFI), adjusted GFI (AGFI), normed fit index (NFI), non-NFI (NNFI) and comparative fit index (CFI) values are all greater than 0.90, and root mean square error of approximation (RMSEA) < 0.05, indicating that the model was well adapted to the actual data.

**Table 3:** Fit index of influencing factors pathway analysis of clinical ability

**Indicators**	**GFI**	**AGFI**	**X**^**2**^	**X**^**2**^**/df**	**NFI**	**NNFI**	**RMSEA**	**CFI**
Correction	0.988	0.961	15.213	1.383	0.973	0.992	0.035	0.992
Reference Value	>0.90	>0.90		<3	>0.90	>0.90	<0.05	>0.90

**Fig. 1:**
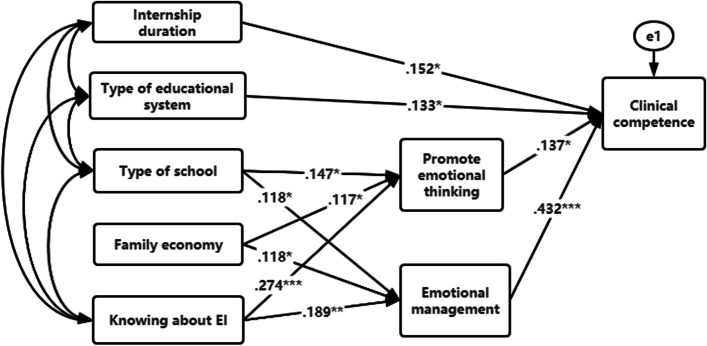
Multivariate path analysis model. * *P *< 0.05, **P = 0. 01, *** *P* < 0.01. The one-way arrow in the figure indicates the relationship from cause to effect.

Path analysis in the structural equation model can quantitatively describe the inherent laws of variables, and study direct and indirect effects among variables. The model includes not only observable explicit variables, but also unobservable latent ones. In the model of Fig. 1, the two dimensions of EI, namely the promotion of emotional thinking and emotional management, have direct effects on clinical ability. In the meantime, it is also influenced by the type of educational system, family economy and the knowledge of EI in the general data of latent variables, which has an indirect effect on clinical ability. The causal relationship and correlation degree among the variables were expressed by a variety of path coefficients, and the influence mechanism among the variables is shown in Fig. 1.

In the process of model building, *P* > 0.05 indicated that the influence of variables on clinical ability failed to reach a 0.05 significant level and was gradually eliminated in the process of model building to build an adaptation model. The maximum likelihood method was adopted to estimate the values of each path coefficient. Eight significant pathways were produced by analyzing the parameters in Fig. 1 and after eliminating the insignificant paths in the model. Among them, the two dimensions of EI, namely the promotion of emotional thinking and emotional management, reached a significant level of clinical ability. Standardized regression coefficients are 0.137 and 0.432, respectively (*P* < 0.05), and corresponding diameter effect values were obtained (see Fig. 1 for details). The influence effect of emotional management on clinical ability was greater than that of the promotion of emotional thinking.

## Discussion

Based on the final fitted model, “managing emotions” and “facilitating thought” of EI branches have direct effects on the clinical ability of nursing interns. Furthermore, the type of school, family financial state and the knowledge of EI indirectly influence clinical ability through their impact on “managing emotions” and “facilitating thought” of EI. In addition, the results showed that internship duration and the educational system also have direct effects on clinical ability.

### Path to the clinical ability of nursing interns

In this study, the EI measurement (ESI) based on the ability EI model contained four branches of ability. It is worth noting that only two branches of EI positively predicted clinical ability. This is a little different from the results of previous studies which showed that clinical practice was positively predicted by total EI scores [22, 23]. Cognitive scientists have noticed that emotions prioritize thinking. Nursing care is a process which is accompanied by critical thinking and clinical decision making, and where emotions are of particular importance to regulate cognition and behavior. According to the ability EI model, “facilitating thought” is the ability of emotions to help thinking in three ways: signaling important environmental changes, changing mood helping individuals to see a situation in several different ways, and assisting in different types of reasoning by means of facilitation [8]. This is consistent with the ability a nurse must be equipped with: observation, critical thinking and clinical decision making through multi-directional clinical reasoning. Existing studies have indicated a positive correlation between the EI and critical thinking or decision making of nursing students and nurses [5, 11].

The “managing emotions” branch has the greatest impact on the clinical ability and involves the ability to manage one’s own emotions and those of people around him [8], influencing the process and time of possessing emotions, and the way in which individuals decide to experience and express emotions [24]. In other words, this branch involves making use of emotions, which is the ability to integrate the other three branches in order to achieve the goal and perform the desired action. Clinical ability is more action-orientated. Every nursing behavior needs corresponding emotions involved. Therefore, it is very important for nurses/nursing students to manage the emotions of themselves and their clients to achieve nursing goals. Further, emotion control can be conducive to interrupting negative emotional states and prolonging positive emotional capacity when an individual feels a negative emotion [25]. The ability of interns to self-regulate their emotions, increase their emotional flexibility, and effectively relieve negative moods such as depression, procrastination and anxiety through positive emotions significantly improves their clinical ability [26, 27].

The finding of this research showed that school type has an indirect impact on clinical ability by influencing EI, and the educational system has a direct impact on clinical ability. That is to say, junior college interns tend to have higher EI, while undergraduate ones tend to have the higher clinical ability. This difference may be related to educational curriculum and learning years. In general, Chinese public universities offer more deferent courses and activities, including elective courses, thereby providing nursing students with more opportunities to engage in social and emotion learning (SEL). Similarly, undergraduate students have more time to learn and experience compared with three-year junior college ones.

The findings also showed that interns from better family economic states are inclined to have higher EI, which was supported by some studies carried out in western countries. Deveney et al. [28] reported that individuals with high socioeconomic status (SES) have higher EI, and Schmalor [29] reported that subjective economic inequality is associated with decreased EI ability, which was not supported by literature in China. It may be related to the investment amount of families in general literacy education.

In addition, the results suggested that interns knowing about EI tend to have a high level of EI, which may just prove that EI can be improved through one’s own learning and self-reflection. Furthermore, it was found that the length of the internship is significantly positively related to the clinical ability of nursing students. This result is in line with several previous studies [14, 24], suggesting that clinical ability changes progressively over time and is graded.

### Levels of EI and clinical ability of nursing interns

In this study, the overall EI of nursing interns was at an upper-moderate level, which is consistent with the results of the study by Štiglic et al. [30]. However, research by Marvos, et al. [31] showed that the EI of most nursing students is at an intermediate level, which can be explained by the fact that the subjects were all undergraduates and included nursing students of all grades.

Research objects included undergraduates and junior college students who were all nurse interns. Among the four branches of EI, the highest branch was the degree of “managing emotions”, indicating that nursing students with high EI had formed positive psychological qualities through a good emotional experience, generated positive emotions in negative situations and maintained them for a long time [32]. Nursing students with high EI are also more empathetic, inclined to be better at establishing good relationships with patients and their families, and able to manage their emotions more effectively [33]. The lowest EIS score observed in this study was for the ability to “understand emotions”, indicating that the interns included in this study were less capable of recognizing and analyzing their own emotions and those of others. Besides, Cheshire et al. [34] showed that the ability of nursing students to understand emotions is low, and will decline further with the passage of time. Interns have good communication skills and empathy, whose understanding of non-verbal communication and communication forms however remains incomprehensive. As a consequence, they can neither properly convey their emotions through non-verbal communication nor understand and analyze the emotions conveyed by other people in nonverbal messages.

The mean score of the overall clinical ability of interns represented an upper-middle level, indicating that their clinical ability was affirmed. This is consistent with the results of Farshi et al. [35]. Notarnicola et al. [12] also showed that most nursing students consider their clinical ability "good" and themselves competent for nursing work. However, another study [36] revealed that nursing students who have just graduated or are about to graduate have acceptable clinical ability but lack certain clinical skills and a systematic understanding of clinical nursing, with relatively fragmented clinical knowledge. This may be due to interns have not been independent in their clinical practice, but simply complete clinical internship tasks and neglect their professional development without paying attention to learning, thinking and reflection. Nevertheless, learning and reflection are the cornerstones of the development of professional ability in the internship process, and learning clinical skills is essential for nurses to develop their ability and perform their work [37]. The results of the present study also showed that the learning of clinical operating skills and knowledge of nursing trainees has not yet formed a system, and thus the professional development dimension of the clinical ability of nursing interns got the lowest score.

### Implications for nursing education

In recent decades, EI has attracted much attention, which is partly due to the idea that EI can be learned and developed by education. The findings from this study provided valuable evidence to support the predicting effects of EI on the clinical ability of nursing interns. It was suggested that nursing colleges should design and implement EI courses into the curriculum with the goal of strengthening the EI of nursing students and ultimately their clinical ability when nursing students study at school. In the design of these training, attention should be paid to assessing the level of EI branches of students, especially “managing emotions” and “facilitating thought”, and then developing targeted training programs. Simulation, practical and team-based learning (TBL) [38–40] which addresses how to improve clinical ability from the perspective of emotions by improving the EI level of students, can be considered for flexible use. In addition, reflection is increasingly being advocated as a strategy for developing EI. Additionally, it is also necessary to take into consideration the background of students and the conditions of schools. Good social support, emotions and adaptability to campus life can improve the level of EI in nursing education [41].

### Strengths and limitations

This study is the first to explore the impact of EI on the clinical ability of nursing interns with path analysis in mainland of China. The finding might provide meaningful implications for nursing education in the Chinese mainland and other countries. However, this study has several limitations. Firstly, the samples were recruited from only one tertiary hospital in Shanghai, China. Therefore, the generalizability of findings may be limited. Secondly, the cross-sectional design was used, limiting the assessment of the impact of EI on clinical competence. Lastly, self-report measures were solely used to assess EI and clinical ability in the clinical context. Objective measures are needed in the future.

## Conclusion

EI plays an essential part in nursing education. This study explored the influence of EI on the clinical ability of nursing interns through an SEM analysis and suggested how this relationship was produced from mediating variables. In particular, different branches of EI, their impact on clinical ability as well as the mediating effect of background factors were discussed as well. Based on these findings, it was suggested that EI training should be integrated into the whole nursing education, and intervention could focus on the roles of facilitating thought and managing emotions of EI. In addition, educators need to consider the context of nursing students and the characteristics of schools. In the future, intervention research like randomized controlled trials (RCTs) should be carried out to test the effects of EI training programs.

## Supplementary Information

**Additional file 1.** 

## Data Availability

The datasets used and analyzed during the present study are available from the corresponding author on reasonable request。

## Abbreviations

EI: Emotional Intelligence
EIS: Chinese version of emotional intelligence scale
HCAT: Chinese Version of Overall Clinical Ability Assessment Tool
TBL: team-based Learning Method
